# Impact of Anticipated Awake Surgery on Psychooncological Distress in Brain Tumor Patients

**DOI:** 10.3389/fonc.2021.795247

**Published:** 2022-01-17

**Authors:** Franziska Staub-Bartelt, Oliver Radtke, Daniel Hänggi, Michael Sabel, Marion Rapp

**Affiliations:** Department of Neurosurgery, University Hospital Duesseldorf, Duesseldorf, Germany

**Keywords:** awake surgery, psychooncological distress, glioblastoma, brain tumor, HADS, EORT C QLQ-C30

## Abstract

**Background:**

Brain tumor patients present high rates of distress, anxiety, and depression, in particular perioperatively. For resection of eloquent located cerebral lesions, awake surgery is the gold standard surgical method for the preservation of speech and motor function, which might be accompanied by increased psychological distress. The aim of the present study was to analyze if patients who are undergoing awake craniotomy suffer from increased prevalence or higher scores in distress, anxiety, or depression.

**Methods:**

Patients, who were electively admitted for brain tumor surgery at our neurooncological department, were perioperatively screened regarding distress, anxiety, and quality of life using three established self-assessment instruments (Hospital Anxiety and Depression Scale, distress thermometer, and European Organisation for Research and Treatment of Cancer (EORTC) QLQ-C30-BN20). Screening results were correlated regarding operation technique (awake vs. general anesthesia). Retrospective statistical analyses for nominal variables were conducted using chi-square test. Metric variables were analyzed using the Kruskal–Wallis test, the Mann–Whitney U-test, and independent-samples t-tests.

**Results:**

Data from 54 patients (26 male and 28 female) aged 29 to 82 years were available for statistical analyses. A total of 37 patients received primary resection and 17 recurrent tumor resection. Awake surgery was performed in 35 patients. There was no significant difference in awake versus non-awake surgery patients regarding prevalence (of distress (p = 0.465), anxiety (p = 0.223), or depression (p = 0.882). Furthermore, awake surgery had no significant influence on distress thermometer score (p = 0.668), anxiety score (p = 0.682), or depression score (p = 0.630) as well as future uncertainty (p = 0.436) or global health status (p = 0.943). Additionally, analyses revealed that primary or recurrent surgery also did not have any significant influence on the prevalence or scoring of the evaluated items.

**Conclusion:**

Analyses of our cohort’s data suggest that planned awake surgery might not have a negative impact on patients concerning the prevalence and severity of manifestation of distress, anxiety, or depression in psychooncological screening. Patients undergoing recurrent surgery tend to demonstrate increased distress, although results were not significant.

## Introduction

Cancer patients are at high risk of suffering increased levels of distress, anxiety, and depression. A study regarding the prevalence of distress in patients with different types of cancer reported an overall prevalence of distress of about 35% (1). When focusing on neurooncological patients, prevalence of distress is reported to be even higher with ranges of between 38% and 52% (2, 3). According to previous studies, approximately one-fourth of cancer patients also suffer from depression or depressive symptoms (4). In brain tumor patients, the prevalence of depression is reported to be approximately 21% and generally assumed to be higher than in patients with different cancers (4, 5). Further analyses underlined that in correlation to increased levels of distress, anxiety, and depression, brain tumor patients additionally show a reduction of quality of life (QoL) (6, 7), finally resulting in decreased overall survival. Studies reported that psychological distress is associated with increased cancer mortality (8) and significantly worse outcomes in cancer patients with brain tumors, especially in patients with high-grade glioma (9–12). Longitudinal analyses regarding distress in neurooncological patients underlined increased distress especially perioperatively during hospitalization (13). Therefore, in particular, perioperative screening to facilitate a timely additional psychooncological support seems to be crucial.

The aim of surgery in neurooncological patients is a maximal aggressive tumor resection without causing permanent neurological deficits. In order to achieve this goal, the operation techniques were significantly improved by using neuronavigation, fluorescence-guided surgery, and intraoperative neuromonitoring during the last decade. Especially awake surgery in patients with eloquent located lesions has been proven to maximize the extent of resection leading to an improved outcome while decreasing risks for new postoperative neurological deficits (14–16). But less is known if this anticipated operation technique causes additional distress for neurooncological patients.

Therefore, the present study aimed to answer the question of whether the anticipation of awake surgery has an additional negative impact on distress, anxiety, depression, and QoL status in neurooncological patients in the preoperative phase as compared with patients undergoing surgery under general anesthesia (GA).

## Patients and Methods

In this retrospective single-center analysis (screening period January 2019 to September 2020), we investigated the perioperative impact of anticipation of awake surgery regarding psychooncological distress of brain tumor patients. The study was approved by the local ethics committee (Study Number 4087). Reporting of this study was according to the Strengthening the Reporting of Observational Studies in Epidemiology (STROBE) guidelines for observational studies (**Supplementary Material**).

### Patients

Inclusion criteria for the present analysis were 1) patients age >18 years with the diagnosis of a brain tumor 2) who were electively admitted for tumor surgery at our neurooncological department with 3) a complete preoperative data set of distress and QoL assessment. Due to the retrospective study design, assessment questionnaires were filled out quite heterogeneously with partially missing data. In order to avoid interference of analyses by an indifferent amount of data for each single screening parameter, we defined that only patients with a complete psychooncological screening assessment were eligible for inclusion. Patients with missing data in any of the below-described screening items were excluded, finally leading to exclusion of 74.65% of the patients. Screening assessment comprising all screening items will be described further below.

**Figure 1:**
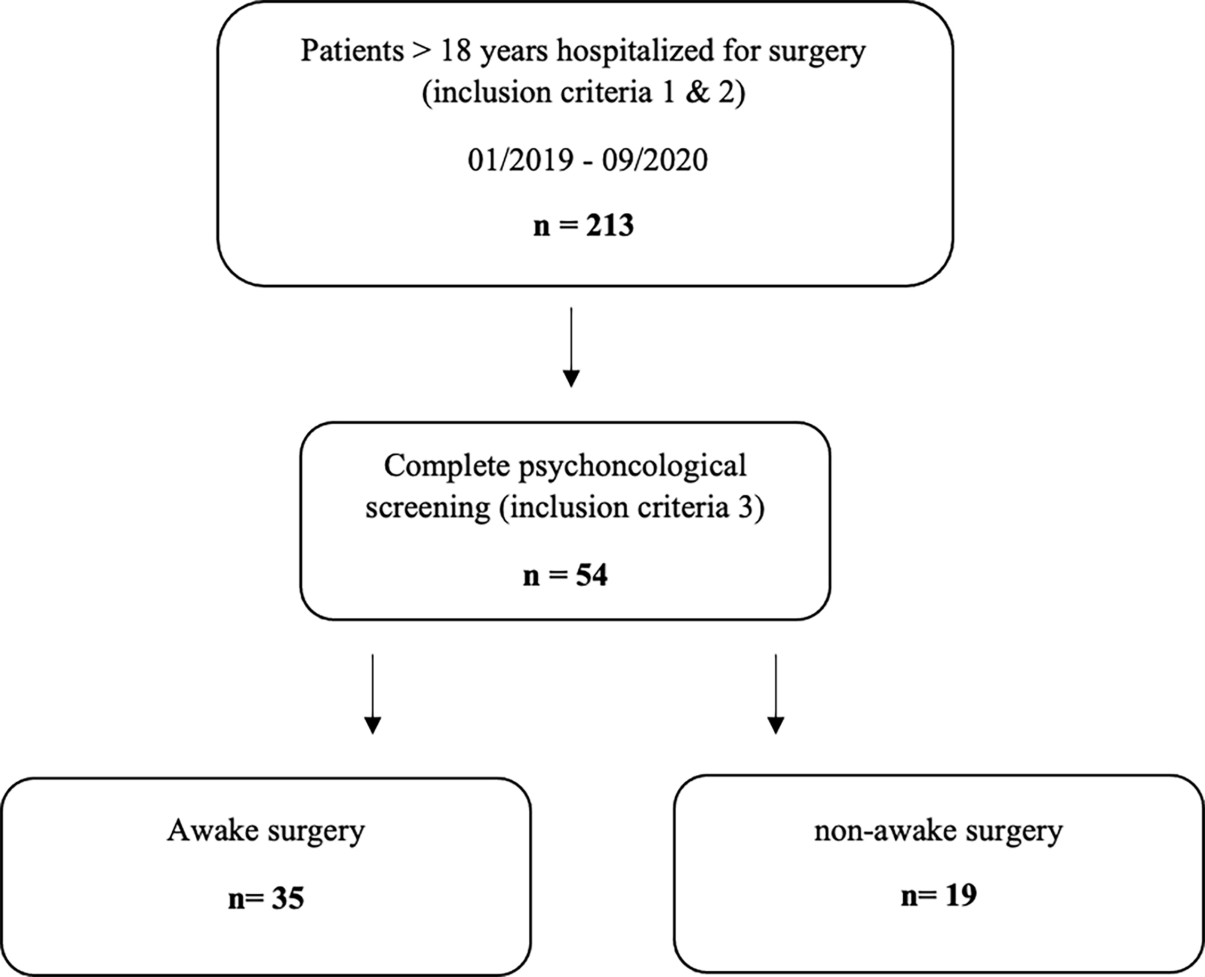
Flowchart describing patient selection.

For further analysis, patients were divided regarding their resection modality (awake vs. GA) (**Figure 1**). Secondly, analyses concerning the impact of primary or recurrent surgery were performed.

Detailed epidemiological information including clinical data of both groups is summarized in **Table 1**.

**Table 1:** Descriptive epidemiologic data of patient cohort.

	Awake (n = 35)	Non-awake (GA) (n = 19)
Age (years)		
Mean	55 [SEM ± 2.9]	59 [SEM ± 2.8]
Range	29–81	40–83
Gender		
Female	21	7
Male	14	12
Diagnosis		
Glioblastoma (WHO IV)	18	13
Anaplastic oligodendroglioma (WHO III)	2	0
Anaplastic astrocytoma (WHO III)	3	1
Diffuse glioma (WHO II)	2	0
Astrocytoma IDH mutant (WHO II)	5	0
Cerebral metastases	4	2
Cerebral lymphoma	0	3
Ganglioglioma (WHO I)	1	
ECOG pre-op		
Mean	0.8 [SEM ± 0.1]	1.0 [SEM ± 0.2]
ECOG post-op		
Mean	0.9 [SEM ± 0.2]	1.2 [SEM ± 0.2]
Primary surgery	25	12
Recurrent surgery	10	7

GA, general anesthesia; ECOG, Eastern Cooperative Oncology Group.

### Screening Assessments

Screening was performed as tablet-based self-assessment after short instruction by our medical staff. All patients were screened 1–2 days preoperatively during hospitalization with the following instruments.

#### National Comprehensive Cancer Network, Distress Thermometer

As a routine screening instrument for distress in cancer patients, the distress thermometer (DT) was firstly published in 1999 by the National Comprehensive Cancer Network (NCCN). The DT is now part of the NCCN guidelines and an easy-to-administer self-reporting tool with a rating scale ranging from 0 (no distress) to 10 (extreme distress). According to the NCCN guidelines, we defined a DT score of 5 or above as indicating distress. The DT also contains a list of 40 symptoms representing practical, family, emotional, spiritual–religious, and physical concerns. In our setting, we only used the visual scale, and the symptom list was excluded.

#### Hospital Anxiety and Depression Scale

Firstly published in 1983, the Hospital Anxiety and Depression Scale (HADS) was originally designed to assess the psychological state of physically ill patients. Meanwhile, it has been established as an effective screening tool for the assessment of anxiety and depression. The 14-item self-report questionnaire consists of 7 items used to identify anxiety (HADS-A) and 7 items for depression (HADS-D), with each item having a 4-point (0–3) Likert-type scale. The maximum score on each subscale is 21 points. A cutoff score of >8 is assumed to be optimal concerning sensitivity and specificity in defining anxiety disorders in patients (17, 18).

#### Health-Related Quality of Life Assessment

The European Organisation for Research and Treatment of Cancer (EORTC) QLQ-C30-BN20 is a disease-specific questionnaire developed by the EORTC to assess the QoL of cancer patients. The EORTC QLQ-C30 consists of a 4-point scale containing four function scales, three symptom scales, and six single-item scales as well as two 7-point scales: the global health status and the QoL. The QLQ-BN20 is an additional module for brain tumor patients, consisting of 20 questions specifically assessing brain tumor-related symptoms. Distress screening results were correlated with the following items: global health status, QoL, and future uncertainty (7, 19). The threshold for the global health and QoL score was ≤4 and for emotional function, cognitive function, and future uncertainty ≥2.75, scored according to the recommended scoring manual of the EORTC.

### Indication for Awake Surgery and Preparation Protocol

For eloquent (cortically and/or subcortically) located tumors (evaluated in preoperative MRI scans), awake surgery with intraoperative monitoring was planned to preserve functionality. In patients with suspected language affecting lesions or for specific motoric testing, awake surgery was indicated in order to perform adequate intraoperative monitoring of function (14, 20). Speech monitoring was performed using 60-Hz stimulation, and motor stimulation was performed using high-frequency monopolar stimulation.

Independently from localization, patients with severe preoperative speech disorders were excluded from the awake surgery group.

All patients in the awake group underwent baseline testing 1 day prior to surgery, with the same tests used intraoperatively. Additionally, the intraoperative setting of awake surgery was practiced with the patients in order to prepare patients for the upcoming procedure.

In patients with a suspected malignant brain tumor, 5-aminolevulinic acid (5-ALA) was administered orally 3–4 h prior to surgery. 5-ALA leads to the accumulation of fluorescent porphyrins in malignant cells and helps intraoperatively with the identification of tumor tissue leading to the increased extent of resection and increased progression-free survival in patients with malignant glioma (21, 22).

### Statistical Analyses

Obtained results were statistically analyzed by using the chi-square test for nominal variables. Metric variables were analyzed using the Kruskal–Wallis test, the Mann–Whitney U-test, and independent-samples t-tests. Statistical analyses were conducted using IBM SPSS Statistics Version 26 (IBM Corporation, USA). Statistical cutoff stated as p-value was set at 0.05.

## Results

Fifty-four out of 213 patients were eligible for inclusion in the final analysis (**Figure 1**). Of the patients, 26 were male and 28 female, with mean age of 56.04 [ ± 2.1 SEM]. A total of 37 patients (68.52%) received first tumor resection, and 17 (31.48%) were hospitalized due to recurrent surgery. Out of 54, 35 patients were undergoing awake surgery (64.81%), as intraoperative speech and motoric testing were required for enabling safe resection due to eloquent localization of the lesion. In the recurrent patient group, there was one case where surgical procedures had changed (primary surgery, non-awake; recurrent surgery, awake surgery). Sixteen patients underwent recurrent surgery following the same surgical strategy compared with primary surgery. The subgroups’ mean time between primary and recurrent surgery was 2.9 years [ ± 0.54 SEM].

Preoperative mean Eastern Cooperative Oncology Group (ECOG) Performance Status was 0.9 [ ± 0.1 SEM] and postoperative 1 [ ± 0.1 SEM]. Neither of the included patients reported a psychiatric diagnosis in medical history.

### Screening Results

Independent from the screening instruments, in the awake patient cohort, 22 patients were indicated to suffer from increased distress (62.86%). In comparison, 10 out of 19 patients who were undergoing surgery under GA complained about distress (52.63%). The prevalence of distress (p = 0.465) did not significantly differ between both cohorts. Furthermore, six patients of the awake patient cohort indicated increased anxiety (17.14%) and five depression (14.29%). Also, six patients (31.58%) in the GA cohort (n = 10/19) reported anxiety and three depression (15.79%). Therefore, again, the prevalence of anxiety (p = 0.223) and depression (p = 0.882) did not differ significantly between patients who were undergoing awake surgery and patients undergoing surgery under GA.

The main results are presented regarding the different screening instruments.

#### Distress Thermometer

**Figure 2:**
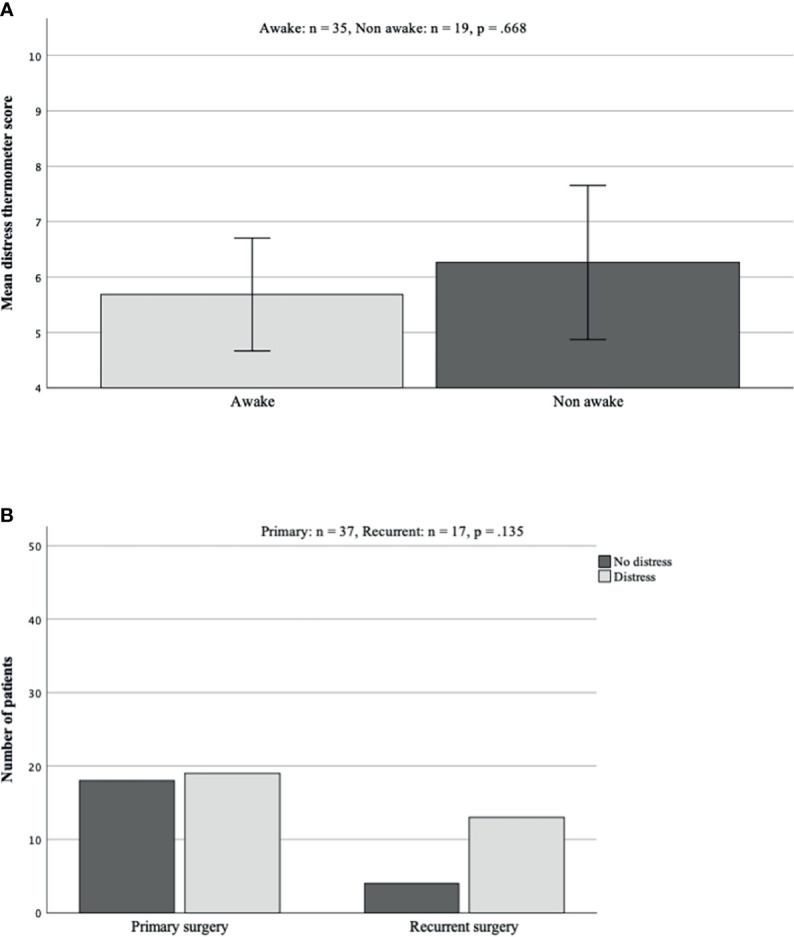
**(A, B)** Mean scoring in DT in group comparison. There were no significant differences seen between both patient groups (**A**; p = 0.668). Concerning comparison of reported distress in either primary or recurrent surgery, although not statistically significant, patients undergoing recurrent surgery reported distress more often **(B)**. DT, distress thermometer.

Regarding results of the DT assessment, the mean score in the awake surgery patient cohort was 5.69 [ ± 0.50 SEM], compared with 6.26 [ ± 0.66 SEM] for patients undergoing surgery under GA. Statistical analyses revealed no significant difference in the scoring of DT in both cohorts (p = 0.668, **Figure 2A**).

Regarding the impact of recurrent surgery, there was no significant influence, although patients undergoing recurrent surgery tended to demonstrate increased distress more often (recurrent 76.47% vs. primary 51.35%, **Figure 2B**).

#### Hospital Anxiety and Depression Scale

**Figure 3:**
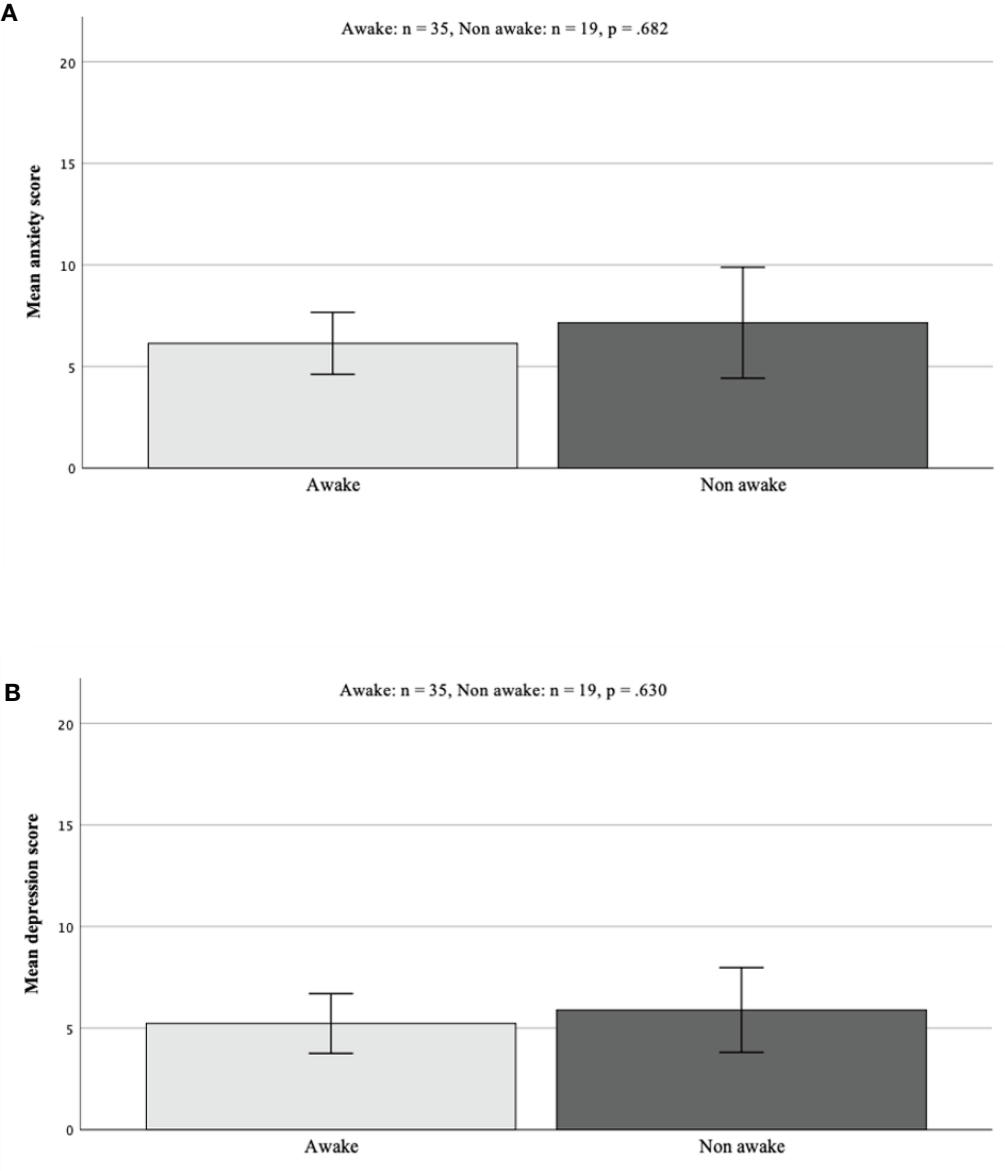
**(A, B)** Group comparison of scores in HADS screening. Results did not differ significantly [awake vs. GA anxiety scores p = 0.682 **(A)**; depression scores p = 0.630 **(B)**]. HADS, Hospital Anxiety and Depression Scale; GA, general anesthesia.

Scoring of anxiety and depression items showed a mean score of 6.14 [ ± 0.75 SEM] for anxiety and 5.23 [ ± 0.72 SEM] for depression score in the awake group. In comparison, the mean score in the GA group was 7.16 [ ± 1.29 SEM] for anxiety and 5.89 [ ± 0.99 SEM] for depression. Neither of both results reached significance (anxiety awake vs. GA p = 0.682; depression p = 0.630, **Figures 3A, B**).

Comparable with the DT results, although recurrent surgery had no significant influence on the prevalence or scoring of both parameters, patients with recurrent surgery tend to demonstrate higher scores for anxiety and depression.

#### Quality of Life

**Figure 4:**
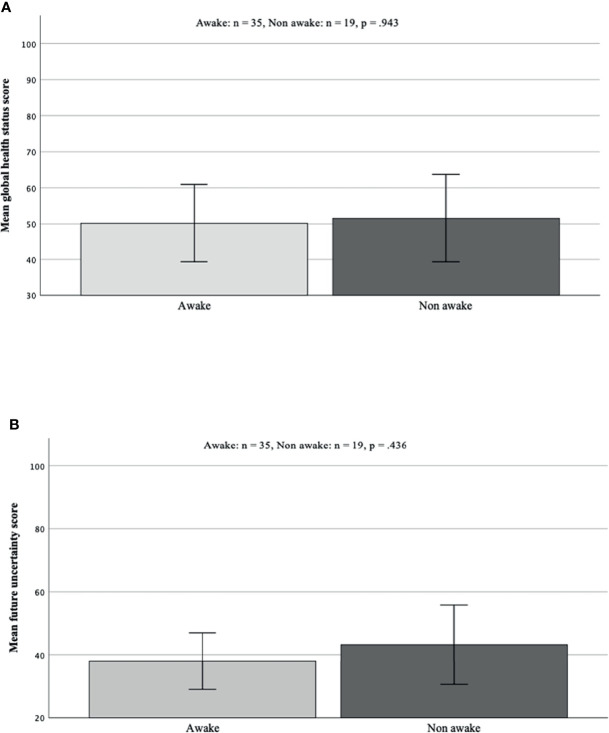
**(A, B)** Analysis of differences in future uncertainty and global health status taken from *EORTC QLQ-BN20* quality of life questionnaire. There was no significant difference in mean scores in both analysed items [global health status p = 0.943 **(A)** future uncertainty p = 0.436 **(B)**]. EORTC, European Organisation for Research and Treatment of Cancer.

Concerning analyses of global health status and future uncertainty from the EORTC brain module, awake surgery did not have any significant influence on scores of future uncertainty (p = 0.436) or global health status (p = 0.943, **Figures 4A, B**).

Again, there was no significant impact on those parameters concerning recurrent surgery; however, a decreased global health status was observed in the recurrent surgery patient cohort.

#### Psychooncological Support

At hospitalization, all patients were asked if they wish to get psychooncological support perioperatively. Fifteen patients (27.78%) accepted additional support. Regarding different patient groups awake vs. GA and recurrent vs. primary surgery, there was no difference (p > 0.05).

## Discussion

For eloquent located tumors, awake surgery is the gold standard treatment option to obtain maximal safe resection. But less is known about the potential negative impact that this additional pressure may have on neurooncological patients who are already at high risk for increased distress, anxiety, and depression.

We aimed to include various essential psychooncological testing parameters in order to obtain a comprehensive overview of the preoperative psychological status of patients when undergoing awake surgery compared with undergoing surgery under GA. Our data reflect that patients undergoing awake surgery for cerebral lesions do not demonstrate distress, anxiety, or depression more often than patients who are undergoing surgery under GA in the preoperative phase. Furthermore, our analysis also clearly underlines the additional impact of recurrent surgery regarding increased distress.

There are only a few data regarding the psychooncological impact of anticipated awake surgery. Ruis et al. analyzed 70 patients using the HADS, and they reported a mean anxiety scale of 6.1 points, comparable with our data. In this analysis, particularly younger patients and women were identified with higher anxiety scores (23). However, they did not compare results with patients undergoing surgery under GA. Our 2013 research group performed a postoperative survey of brain tumor patients who received awake surgery. Most patients stated that they would undergo awake surgery at any time again. A thoroughly pre-op preparation was the most important to support the patients in this situation (24). Different studies underlined that detailed preparation of a well-selected patient cohort is essential to prevent stress disorders and negative psychological aftereffects (25, 26). In this context, Santini and colleagues firstly reported psychological profiling for candidates of awake surgery under the use of psychological questionnaires, neuropsychological testing of language, neurocognition, and intraoperative interviews (27).

At our department, patients undergo a preoperative psychooncological screening as described before; furthermore, a simulation of the awake situation 1 day before scheduled surgery is performed, and all intraoperative performance tasks are explained and practiced with the patient.

Besides careful patient selection and preparation, participation in the decision making and anticipated active role throughout surgery and therefore active role in a positive surgery outcome might contribute to the fact that awake surgery does not have a negative influence on the patients (28). Additionally, contrary to our preoperative screening results, published reports of postoperative screening results of patients undergoing awake craniotomy also revealed no major negative impact of awake surgery on patients. Goebel et al. described pre- and postoperative HADS anxiety and depression scores of 25 patients undergoing awake surgery combined with intraoperative MRI, and only 1 patient showed negative reaction to surgery protocol postoperatively (29). In line with that, Danks et al. reported no major consequences like post-traumatic stress disorders after awake surgery (29, 30).

Although not significant, independent from the assessment instrument, here, patients undergoing recurrent surgery presented increased scores for distress, anxiety, and depression. In the literature, there are some data about distress in the course of neurooncological diseases. There seem to be specific time points, where increased distress was observed, especially during hospitalization as well as at the time point of tumor recurrence (31). This might be due to various general apprehensions when being diagnosed with recurrent cancer. In our cohort additionally, a worse subjective global health status at recurrence was revealed, and that might have also contributed to the increase of psychooncological screening scores.

However, along with positive results from our study, we also have to state major limitations of our data analyses with an arguable small cohort due to our restrictive inclusion criteria. We only included patients with full data sets during preoperative psychooncological testing in order to generate comparability in all analyzed categories. That led to exclusion of approximately 75% of the screened patients. Furthermore, the size of both patient groups quite differed in numbers and might have led to some bias in the analysis.

Nevertheless, to our knowledge, this is the first analysis of a comprehensive psychooncological screening in patients undergoing brain tumor surgery under either awake or non-awake surgery. Hence, our data are of high importance, as awake surgery offers a full range of intraoperative monitoring of speech and motor function for the surgeon, which is essential in patients with brain tumors of some locations. According to previous research perioperative psychooncological distress, anxiety and depression can have a negative influence on the outcome and the patients’ subjective perception of global health care status, which is quite the opposite of the treatment intention. Therefore, an indication for awake surgery has to be questioned for every single patient. But our data show that with detailed preparation and close monitoring of the patients, awake surgery does not have any negative influence on patients, and we can expect our patients to go through this procedure without harming them. On the contrary, the positive effects of a possible increased extent of resection and that accompanying increased overall survival predominate.

## Conclusion

Our data demonstrate that anticipation of awake surgery represents no significant impact for increased distress, anxiety, or depression preoperatively. Surgeons can expect their patients to undergo awake surgery without increasing psychooncological distress. If expected localization of cerebral lesion includes eloquent areas, awake surgery is recommended in order to increase the safety of the patient.

Even if the results were not significant, our data clearly illustrate that patients undergoing recurrent surgery tend to demonstrate increased distress; in this special situation, early contact with professional psychooncologists is recommended.

## Data Availability Statement

The raw data supporting the conclusions of this article will be made available by the correspondent author on reasonable request.

## Ethics Statement

The studies involving human participants were reviewed and approved by the ethics committee of the medical faculty of the University of Düsseldorf (Study Number 4087). The patients/participants provided their written informed consent to participate in this study.

## Author Contributions

MR designed and directed the project. FS-B analyzed the data and wrote the paper. OR analyzed the data. DH and MS provided critical feedback and helped shape the research and manuscript. All authors discussed the results and commented on the manuscript.

## Conflict of Interest

The authors declare that the research was conducted in the absence of any commercial or financial relationships that could be construed as a potential conflict of interest.

## Publisher’s Note

All claims expressed in this article are solely those of the authors and do not necessarily represent those of their affiliated organizations, or those of the publisher, the editors and the reviewers. Any product that may be evaluated in this article, or claim that may be made by its manufacturer, is not guaranteed or endorsed by the publisher.
